# Self-Medication Practices in Medical Students During the COVID-19 Pandemic: A Cross-Sectional Analysis

**DOI:** 10.3389/fpubh.2022.803937

**Published:** 2022-03-09

**Authors:** Farah Yasmin, Muhammad Sohaib Asghar, Unaiza Naeem, Hala Najeeb, Hamza Nauman, Muhammad Nadeem Ahsan, Abdullah Khan Khattak

**Affiliations:** ^1^Department of Internal Medicine, Dow Medical College, Dow University of Health Sciences, Karachi, Pakistan; ^2^Department of Internal Medicine, Dow University of Health Sciences–Ojha Campus, Karachi, Pakistan; ^3^Department of Nephrology, Dow University of Health Sciences–Ojha Campus, Karachi, Pakistan; ^4^Department of Internal Medicine, Aga Khan University Hospital, Karachi, Pakistan

**Keywords:** self-medication, medical students, COVID-19, pandemic, public health, Pakistan

## Abstract

**Background and Objectives:**

During the pandemic, the growing influence of social media, accessibility of over-the-counter medications, and fear of contracting the virus may have led to self-medication practices among the general public. Medical students are prone to such practices due to relevant background knowledge, and access to drugs. This study was carried out to determine and analyze the prevalence of self-medication practices among medical students in Pakistan.

**Materials and Methods:**

This descriptive, cross-sectional study was conducted online in which the participants were asked about the general demographics, their self-medication practices and the reasons to use. All participants were currently enrolled in a medical college pursuing medical or pharmacy degree. Non-probability sampling technique was used to recruit participants.

**Results:**

A total of 489 respondents were included in the final analysis. The response rate was 61%. Majority of the respondents were females and 18–20 years of age. Self-medication was quite prevalent in our study population with 406 out of 489 individuals (83.0%) were using any of the drugs since the start of pandemic. The most commonly utilized medications were Paracetamol (65.2%) and multivitamins (56.0%). The reasons reported for usage of these medications included cold/flu, or preventive measures for COVID-19. The common symptoms reported for self-medication included fever (67.9%), muscle pain (54.0%), fatigue (51.7%), sore throat (46.6%), and cough (44.4%). Paracetamol was the most commonly used drug for all symptoms. Female gender, being in 3rd year of medical studies, and individuals with good self-reported health were found more frequent users of self-medication practices.

**Conclusion:**

Our study revealed common self-medication practices among medical and pharmacy students. It is a significant health issue especially during the pandemic times, with high consumption reported as a prevention or treating symptoms of COVID-19.

## Introduction

The Coronavirus 2019 (COVID-19) was declared a pandemic by the World Health Organization on January 30th, 2020 (1). As of October 22nd, 2021, the global crisis has affected 242 million individuals and claimed over 4.9 million lives (2).

With clinical trials of multiple drugs in the pipeline, there are no definitive treatments with confirmed evidence against the severe acute respiratory syndrome-coronavirus-2 (SARS-CoV-2). Anti-malarial drugs, hydroxychloroquine, and chloroquine received emergency approval to treat prophylaxis in COVID-19 when a small non-randomized trial was initially promising (3). It was soon labeled controversial as randomized studies depicted an increased risk-to-benefit ratio (4). However, rapid medical research has developed, and massively rolled out COVID-19 vaccines prevented the adverse effects from symptoms of the disease in affected individuals (5). It is envisaged that vaccine advent and procurement by developed countries will soon create global inequity as middle-to-low income nations struggle to vaccinate healthcare workers and the older population (6). Economic recession in developing countries, like Pakistan has only managed to fully vaccinate less than half of its population (7).

The third wave of coronavirus had peaked, yet Pakistan's overburdened healthcare system reached a 90% occupancy of ventilators and hospital beds (8). Travel restrictions, oxygen shortage, and the risk of contracting the virus from crowded clinics and hospitals have inculcated fear in the public, allowing a nation prone to self-medication to rely more heavily on self-diagnosis (9, 10). Self-medication is the administering of medicines to treat self-recognized symptoms, psychological ailments, and first aid based on prior knowledge, without consulting a physician (11). The growing influence of social media has let individuals explore COVID-related symptoms, pain keywords, and treatments worldwide, as well as in Pakistan, as revealed by an infodemiological study on Google trends (12). Adding to this is the availability of over the counter (OTC) drugs in pharmacies across Pakistan which has been the highest contributor to a self-medicating prevalence of 81% in the populous Karachi. Easily accessible drugs in pharmacies across Pakistan range from analgesics, antibiotics, anti-diarrheal agents, antihistamines, antipyretics, cough-suppressants, vitamins to herbal medicines and homeopathy (10, 13). Though the primary reason was affordability, the greatest driver (47.8%) of self-medication was the belief that the illness was insignificant. This stems from poor health literacy amongst the general public which is easily influenced by the media (10). It is also well noted that preference for self-medication is cultural practice in Pakistan. The positive and negative effects of self-medication of Sanna Makki, a herbal laxative, which was widely used as a COVID-19 cure after mass-circulation on WhatsApp was a well-publicized example (14). The risks attached to self-medicated drugs include incorrect and improper dosage, and antibiotic abuse leading to drug resistance (15).

A trending worldwide practice of self-medication does not have enough literature on an ongoing pandemic, except studies conducted in Peru and Saudi Arabia (16, 17). However, no study reports the prevalence of self-medication in the highly affected medical student population in Pakistan—the future of the healthcare system. It is important to assess self-medication practices among medical students, because they have relevant knowledge, and are more likely to access to prescription as well as OTC drugs. Low tendency to consult health professionals, reliance on the internet for information on background medical knowledge, and treating self-diagnosed illnesses could be factors that promotes self-medication practice among medical students.

The primary objective was to determine and analyze the prevalence of self-medication practices among medical students for respiratory symptoms, from the onset of first COVID-19 symptom or in case of being positively tested for COVID-19. We further assessed the self-medicated drugs used for respiratory problems during the pandemic. A secondary aim was to evaluate the factors associated with self-medication of various drugs among medical students during the COVID-19 pandemic in Pakistan. This will allow the health ministries and educational administrations alike to undertake measures and address the gap of health negligence that can result from self-medication practices.

## Materials and Methods

### Study Design

This descriptive, cross-sectional study was conducted online from January 25th to February 20th, 2021. The design of the study and sample size was modified according to the pilot study carried out from December 11th to December 15th, 2020, under the expertise of professors and doctors. The questionnaire was developed in English, on Google survey, and distributed through social media platforms. The online survey questions were obtained after a thorough literature search and assessing the validity and reliability (16, 17). The responses were made anonymous to maintain confidentiality and reliability. Each participant received an email of their response to avoid duplication of data. Clarification of the contents and the purpose of the study were explained at the start of the survey followed by an informed consent. Data was anonymized and only the lead investigator had access to the responses.

### Development of Survey Questionnaire and Operationalization of Variables

The questionnaire included a total of 11 questions. Seven questions were dedicated to the general demographics including the city of residence, gender, age, marital status, level of education/medical, and comorbid conditions. The next 4 questions were based on self-medication of drugs for prevention and treatment of respiratory symptoms: drug selection, reasoning for self-medication, symptoms they were looking to improve, and if any of the drugs improved those symptoms. The participants were asked if they consulted any physician before starting these medications, were they been using these medications before COVID-19 pandemic, and do they have any comorbid conditions that warrant the use of these medications. All these questions if answered as a No have been considered as self-medication during the pandemic. The drugs listed in the survey included acetaminophen, ibuprofen, azithromycin, hydroxychloroquine, ivermectin, doxycycline, antivirals (lopinavir, ritonavir, remdesivir, and others), cetirizine, multivitamin compounds or any other drug (open question) for respiratory symptoms. All drugs were classified according to the Anatomical Therapeutic Chemical (ATC) (18). This selection of drugs was based on the existing literature, and reports from the local media. COVID-19 can present with a range of symptoms. Frequently reported symptoms were obtained from CDC's guidelines (19). The list of symptoms included fever, fatigue, cough, muscle ache, nasal congestion, sore throat, headache, breathing difficulty, no symptom, and other symptom (open question). Anosmia was omitted as being controversial at the time of inception of this study (16). To assess the reason for drug use, participants had seven options listed as cold/flu, no symptoms, COVID-19 prevention, had COVID-19 symptoms, COVID-19 positive, consume the drug regularly, other reason (open question). The following 5-point Likert scale was used to identify and list the improvement in symptoms if any: improved all symptoms, improved most of the symptoms, improved a few of the symptoms, improved only one symptom, did not alleviate any of the symptoms.

### Sample Size/Sampling Technique

The sample size of the survey was obtained from the pilot study determined using the power analysis. The minimum sample size was 385, with a statistical power of 80% and a confidence interval of 95%. We hypothesized that at least 50% of the participants would practice self-medication, with a 5% margin of error. The formula for sample size estimation was n = N ×/((N-1) E2 + x), where N is the population size and n is sample size estimation. A non-probability sampling technique was used to recruit participants from social media platforms, including WhatsApp, Facebook, and Messenger.

### Inclusion/Exclusion Criteria

Inclusion criteria included completed responses, and the respondent i.e., male or a female medical student pursuing MBBS or Pharm D degree from a medical college in Pakistan, having the ability to understand English, and being technologically adept and have access to Internet. While exclusion criteria included respondents from other than the medical field.

### Ethical Approval

The study received ethical approval from the Institution's Ethics Committee of the Dow University Ojha Hospital. Each participant had the right to withdraw from the study at any time. The possible risks and the purpose of the survey were thoroughly explained. Participants had to provide consent before filling out the questionnaire.

### Data Analysis

Microsoft Excel 2016 was used for data collection and assembled in to Statistical Package for Social Sciences (SPSS) version 25.0 for data analysis. A multivariate analysis estimated the preference of self-medication for the surveyed drugs using the baseline demographics i.e., sex, age, marital status, educational status, region, presence of comorbid conditions, and self-reported health as control variables. Categorical variables were assessed using frequencies and percentages, through univariate analysis. Analytical statistics were performed with 95% confidence intervals (CI) and odds ratios (OR), which were obtained using logistic regression.

## Results

A total of 374 medical and 115 pharmacy students were included in the final analysis. The response rate was 61%. Majority of the respondents were of 18–20 years of age (58.5%). More than three-fourths were females. 34.0% of medical students were in first year, 20.6% in second year, 20.3% in third year, 13.9% in fourth year, and 11.2% were in final year of their medical education. Most respondents were from province Sindh and Punjab (combined 85.7%). Only 4.5% reported previous comorbid conditions, while majority (60.5%) were in good state of self-reported health. While reporting the risk of acquiring COVID-19 infection, 43.8% were unbothered. Another 38.0% admitted mild risk while only 2.9% reported severe risk. 15.3% of participants were already infected with the virus. The socio-demographic characteristics of the participants are described in Table 1.

**Table 1:** Sociodemographic characteristic of medical students during the COVID-19 lockdown (*n* = 489).

	**Variables**	***N* (%)**
Age	18–20 years	286 (58.5)
	21–25 years	192 (39.3)
	>25 years	11 (2.2)
Gender	Male	108 (22.1)
	Female	381 (77.9)
Student	Medical	374 (76.5)
	Pharmacy	115 (23.5)
Medical year (*n* = 374)^*^	1st year	127 (34.0)
	2nd year	77 (20.6)
	3rd year	76 (20.3)
	4th year	52 (13.9)
	5th year	42 (11.2)
State	Sindh	237 (48.5)
	Punjab	182 (37.2)
	Khyber Pakhtunkhwa	61 (12.5)
	Balochistan	9 (1.8)
Comorbidities	Present	22 (4.5)
	Absent	467 (95.5)
Self-reported health	Excellent	114 (23.3)
	Good	296 (60.5)
	Fair	72 (14.7)
	Poor	7 (1.4)
Self-reported risk of acquiring COVID-19	No risk	214 (43.8)
	Already infected	75 (15.3)
	Mild risk	186 (38.0)
	Severe risk	14 (2.9)
Use of medications	Paracetamol	319 (65.2)
	Ibuprofen	142 (29.0)
	Azithromycin	125 (25.6)
	Hydroxychloroquine	43 (8.8)
	Ivermectin	22 (4.5)
	Doxycycline	19 (3.9)
	Cetirizine	136 (27.8)
	Antivirals	35 (7.2)
	Multivitamins	274 (56.0)
	Others	56 (11.4)
Reason for use	Cold/Flu	349 (71.4)
	Used it without having any symptoms	172 (35.2)
	Used it for prevention of COVID-19 infection	212 (43.3)
	Had COVID-19 symptoms and I self-medicated	167 (34.1)
	Had confirmed positive COVID-19 infection and I self-medicated	129 (26.4)
	Consumed regularly for other reasons	196 (40.1)
	Others	155 (31.7)
Symptomatology reported for medicine use	Fever	332 (67.9)
	Fatigue	253 (51.7)
	Cough	217 (44.4)
	Sneezing	199 (40.7)
	Muscle pain/Body aches	264 (54.0)
	Nasal congestion	206 (42.1)
	Sore throat	228 (46.6)
	Anosmia (loss of smell)	113 (23.1)
	Breathing difficulty	120 (24.5)
	Used it even though I had none of the above symptoms	173 (35.4)
Reporting of symptom improvement after medicine use	Alleviated ALL the symptoms	106 (21.7)
	Alleviated MOST of the symptoms	109 (22.3)
	Alleviated A FEW of the symptoms	91 (18.6)
	Alleviated only ONE symptom	85 (17.4)
	Alleviated NONE of the symptoms	98 (20.0)

*Data presented as frequency and percentage*.

* *Data retrieved for medical students only (n = 374)*.

**Figure 1:**
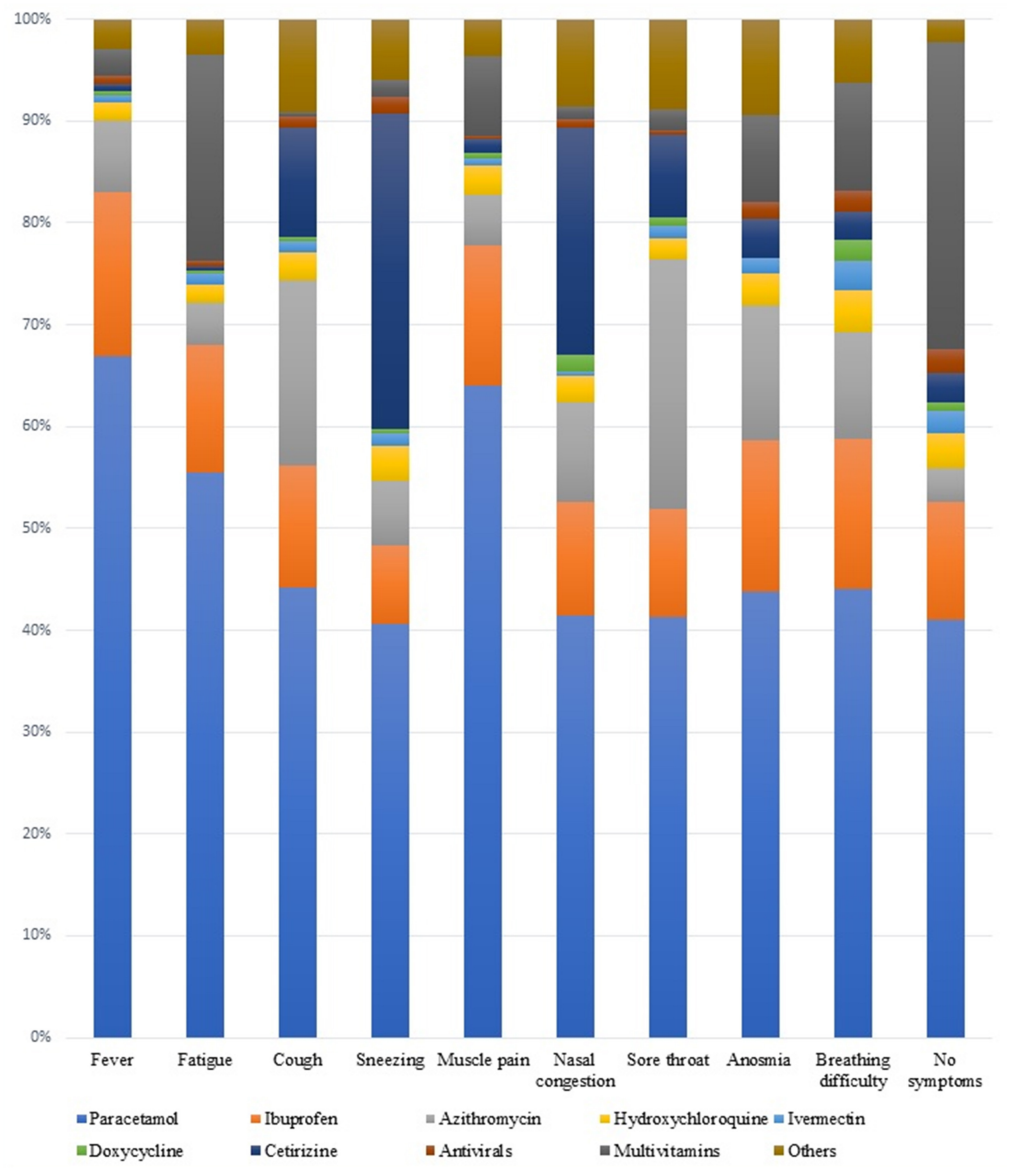
Relative proportions of drug use on specific symptomatology.

Self-medication was prevalent in our study population with 316 out of 374 medical students (84.5%) and 90 out of 115 pharmacy students (78.3%) were using any of the drugs since the start of pandemic. The most commonly utilized medications were Paracetamol (65.2%), multivitamins (56.0%), Ibuprofen (29.0%), Cetirizine (27.8%), and Azithromycin (25.6%) among others. The reasons reported for usage of these medications included cold/flu (71.4%), as preventive measures for COVID-19 (43.3%), or a self-medication for COVID-19 symptoms (34.1%). However, 35.2% reported taking these medications without any reason or symptoms, while another 40.1% consumed them regularly for other reasons. The symptoms reported for self-medication included fever (67.9%), fatigue (51.7%), cough (44.4%), sneezing (40.7%), muscle pain/body aches (54.0%), nasal congestion (42.1%), sore throat (46.6%) anosmia (23.1%), and breathing difficulty (24.5%) as shown in Table 1. Paracetamol was the most commonly used drug for all symptoms including fever, fatigue, cough, sneezing, myalgia, nasal congestion, sore throat, anosmia, and shortness of breath. Figure 1 demonstrates the relative proportions of drug use for each specific symptomatology.

Paracetamol use was common in individuals >21 years, final year medical students, those already infected with COVID-19, and having flu, fever, and fatigue. 45.4% shown all or most symptoms resolved with its use as shown in Table 2. Azithromycin use was common in age >21 years, 5th year medical students, inhabitants of Khyber Pakhtunkhwa/Balochistan, as well as those already infected with COVID-19. Cetirizine use was frequent among pharmacy students, final year medical students and those with self-reported risk of COVID-19 infection. Use of antivirals was prevalent among males of above 21 years of age. Use of multivitamins was also common in individuals >21 years, females, residents of Khyber Pakhtunkhwa/Balochistan and those either already infected with COVID-19 or reported mild to severe risk of acquiring infection. Individuals from province Punjab were more likely to use ivermectin and less likely to use cetirizine as self-medication. Doxycycline use was found prevalent in males. Use of ivermectin was not shown to improve any of the self -reported symptoms. Other medications were more common among those having comorbid conditions and self-reported as fair/poor. All these factors showed significant association when conducted on a logistic regression model with reported odds ratios and *p*-values as shown in Table 3.

**Table 2:** Factors associated with self-medication of various drugs among medical students during the COVID-19 lockdown (*n* = 489).

**Factor**	**Parace-tamol *N* = 319**	**Ibuprofen** ***N* = 142**	**Azithro-mycin *N* = 125**	**HCQ** ***N* = 43**	**Iver-mectin *N* = 22**	**Doxy-cycline** ***N* = 19**	**Cetirizine *N* = 136**	**Antiviral** ***N* = 35**	**Multi-vitamin *N* = 274**	**Other** ***N* = 56**
**Age (years)**										
18–20	155 (48.6)	76 (53.5)	50 (40.0)	26 (60.4)	11 (50.0)	12 (63.2)	65 (47.8)	12 (34.3)	140 (51.1)	32 (57.1)
21–25 and above	**164 (51.4)**	66 (46.5)	**75 (60.0)**	17 (39.5)	11 (50.0)	7 (36.8)	**71 (52.2)**	**23 (65.7)**	**134 (48.9)**	24 (42.9)
**Gender**										
Female	251 (78.7)	114 (80.3)	92 (73.6)	34 (79.1)	18 (81.8)	11 (57.9)	100 (73.5)	20 (57.1)	**233 (85.0)**	43 (76.8)
Male	68 (21.3)	28 (19.7)	33 (26.4)	9 (20.9)	4 (18.2)	**8 (42.1)**	36 (26.5)	**15 (42.9)**	41 (15.0)	13 (23.2)
**Student**										
Medical	244 (76.5)	109 (76.8)	95 (76.0)	34 (79.1)	17 (77.3)	15 (78.9)	95 (69.8)	27 (77.1)	209 (76.3)	44 (78.6)
Pharmacy	75 (23.5)	33 (23.2)	30 (24.0)	9 (20.9)	5 (22.7)	4 (21.1)	**41 (30.1)**	8 (22.9)	65 (23.7)	12 (21.4)
**Medical year**										
1st year	65 (26.6)	28 (25.7)	24 (25.3)	15 (44.1)	5 (29.4)	3 (20.0)	21 (21.6)	6 (22.2)	69 (33.0)	19 (43.2)
2nd year	49 (20.1)	22 (20.2)	15 (15.8)	6 (17.6)	2 (11.8)	3 (20.0)	24 (24.7)	4 (14.8)	40 (19.1)	13 (29.5)
3rd year	56 (23.0)	28 (25.7)	24 (25.3)	7 (20.6)	7 (41.2)	6 (40.0)	22 (22.7)	9 (33.3)	43 (20.6)	5 (11.4)
4th year	40 (16.4)	20 (18.3)	14 (14.7)	2 (5.9)	3 (17.6)	3 (20.0)	15 (15.5)	5 (18.5)	31 (14.8)	5 (11.4)
5th year	**34 (13.9)**	11 (10.1)	**18 (18.9)**	4 (11.8)	0 (0.0)	0 (0.0)	**15 (15.5)**	3 (11.1)	26 (12.4)	2 (4.5)
**State**										
Sindh	154 (48.2)	76 (53.5)	43 (34.4)	19 (44.2)	5 (22.7)	6 (31.6)	71 (52.2)	17 (48.6)	129 (47.1)	26 (46.4)
Punjab	120 (37.6)	45 (31.7)	44 (35.2)	14 (32.6)	**13 (59.1)**	8 (42.1)	**38 (27.9)**	9 (25.7)	96 (35.0)	18 (32.1)
KPK and Balochistan	45 (14.1)	21 (14.8)	**38 (30.4)**	10 (23.2)	4 (18.2)	5 (26.3)	27 (19.9)	8 (22.6)	**49 (17.9)**	12 (21.4)
**Comorbidities**										
Present	13 (4.1)	6 (4.2)	5 (4.0)	3 (7.0)	2 (9.1)	1 (5.3)	6 (4.4)	2 (5.7)	11 (4.0)	**10 (17.9)**
Absent	306 (95.9)	136 (95.8)	120 (96.0)	40 (93.0)	20 (90.9)	18 (94.7)	130 (95.6)	33 (94.3)	263 (96.0)	46 (82.1)
**Self-reported health**										
Excellent	63 (19.7)	33 (23.2)	24 (19.2)	11 (25.6)	2 (9.1)	3 (15.8)	24 (17.6)	8 (22.9)	53 (19.3)	7 (12.5)
Good	204 (63.9)	87 (61.3)	79 (63.2)	24 (55.8)	15 (68.2)	10 (52.6)	83 (61.0)	**15 (42.9)**	176 (64.2)	35 (62.5)
Fair/Poor	52 (16.3)	22 (15.5)	22 (17.6)	8 (18.6)	5 (22.7)	6 (31.6)	29 (21.3)	12 (34.3)	45 (16.4)	**14 (25.0)**
**COVID-19 risk**										
No risk	111 (34.8)	57 (40.1)	44 (35.2)	15 (34.9)	13 (59.1)	11 (57.9)	44 (32.3)	16 (45.7)	92 (33.6)	18 (32.1)
Already infected	**63 (19.7)**	19 (13.4)	**36 (28.8)**	7 (16.3)	2 (9.1)	1 (5.3)	22 (16.2)	2 (5.7)	**68 (24.8)**	11 (19.6)
Mild/Severe	145 (45.4)	66 (46.8)	45 (36.0)	21 (48.8)	7 (31.8)	7 (36.8)	**70 (51.5)**	17 (48.6)	**114 (41.6)**	27 (48.2)
**Reason**										
Cold/Flu	**263 (82.4)**	53 (37.3)	31 (24.8)	10 (23.2)	5 (22.7)	1 (5.3)	53 (39.0)	10 (28.6)	18 (6.6)	27 (48.2)
No symptoms	90 (28.2)	28 (19.7)	16 (12.8)	11 (25.6)	7 (31.8)	1 (5.3)	10 (7.3)	1 (2.8)	81 (29.6)	2 (3.5)
COVID-19 prevention	79 (24.8)	24 (16.9)	21 (16.8)	12 (27.9)	6 (27.2)	2 (10.5)	6 (4.4)	1 (2.8)	71 (25.9)	6 (10.7)
COVID-19 symptoms	104 (32.6)	29 (20.4)	28 (22.4)	7 (16.3)	4 (18.2)	1 (5.3)	7 (5.1)	9 (25.7)	31 (11.3)	13 (23.2)
COVID-19 positivity	92 (28.8)	23 (16.2)	38 (30.4)	6 (13.9)	6 (27.3)	2 (10.5)	5 (3.7)	6 (17.1)	60 (21.9)	16 (28.6)
Regular use	97 (30.4)	21 (14.8)	15 (12.0)	5 (11.6)	4 (18.2)	2 (10.5)	19 (14.0)	5 (14.3)	57 (20.8)	19 (33.9)
Others	105 (32.9)	45 (31.7)	17 (13.6)	12 (27.9)	10 (45.4)	3 (15.8)	15 (11.0)	4 (11.4)	16 (5.8)	9 (16.1)
**Symptoms**										
Fever	**303 (95.0)**	73 (51.4)	32 (25.6)	8 (18.6)	3 (13.6)	2 (10.5)	3 (2.2)	4 (11.4)	12 (4.3)	13 (23.2)
Fatigue	**189 (59.2)**	43 (30.3)	14 (11.2)	6 (13.9)	4 (18.2)	1 (5.3)	1 (0.7)	2 (5.7)	69 (25.2)	12 (21.4)
Cough	112 (35.1)	30 (21.1)	46 (36.8)	7 (16.3)	3 (13.6)	1 (5.3)	27 (19.8)	3 (8.6)	1 (0.3)	23 (41.1)
Sneezing	96 (30.1)	18 (12.7)	15 (12.0)	8 (18.6)	3 (13.6)	1 (5.3)	73 (53.7)	4 (11.4)	4 (1.5)	14 (25.0)
Muscle pain	196 (61.4)	42 (29.6)	15 (12.0)	9 (20.9)	2 (9.1)	2 (10.5)	4 (2.9)	1 (2.9)	24 (8.8)	11 (19.6)
Nasal congestion	97 (30.4)	26 (18.3)	23 (18.4)	6 (13.9)	1 (4.5)	4 (21.1)	52 (38.2)	2 (5.7)	3 (1.1)	20 (35.7)
Sore throat	98 (30.7)	25 (17.6)	58 (46.4)	5 (11.6)	3 (13.6)	2 (10.5)	19 (14.0)	1 (2.9)	5 (1.8)	21 (37.5)
Anosmia	56 (17.5)	19 (13.4)	17 (13.6)	4 (9.3)	2 (9.1)	0 (0.0)	5 (3.7)	2 (5.7)	11 (4.0)	12 (21.4)
Breathing difficulty	63 (19.7)	21 (14.8)	15 (12.0)	6 (13.9)	4 (18.2)	3 (15.8)	4 (2.9)	3 (8.6)	15 (5.5)	9 (16.1)
No symptoms	110 (34.5)	31 (21.8)	9 (7.2)	9 (20.9)	6 (27.3)	2 (10.5)	8 (5.9)	6 (17.1)	81 (29.6)	6 (10.7)
**Alleviated symptoms**										
All of them	**76 (23.8)**	30 (21.1)	24 (19.2)	9 (20.9)	2 (9.1)	3 (15.8)	28 (20.6)	8 (22.6)	59 (21.5)	12 (21.4)
Most of them	69 (21.6)	32 (22.5)	29 (23.2)	9 (20.9)	3 (13.6)	4 (21.1)	30 (22.0)	6 (17.1)	56 (20.4)	9 (16.1)
Few of them	58 (18.2)	26 (18.3)	19 (15.2)	7 (16.3)	2 (9.1)	4 (21.1)	24 (17.6)	7 (20.0)	54 (19.7)	12 (21.4)
One of them	55 (17.2)	25 (17.6)	25 (20.0)	8 (18.6)	3 (13.6)	3 (15.8)	25 (18.4)	5 (14.3)	50 (18.2)	13 (23.2)
None of them	61 (19.1)	29 (20.4)	28 (22.4)	10 (23.2)	**12 (54.5)**	5 (26.3)	29 (21.3)	9 (25.7)	55 (20.1)	10 (17.9)

*Bold values signify significant p-value (<0.05) by either chi-square or Fisher's exact test*.

*Data presented as frequency and percentage*.

*HCQ, hydroxychloroquine; KPK, Khyber Pakhtunkhwa*.

**Table 3:** Bivariate analysis of the factors associated with the self-medication of various drugs during the COVID-19 lockdown among medical students (*n* = 489).

**Variable**	**Parace-tamol**	**Ibuprofen**	**Azithro-mycin**	**HCQ**	**Iver-mectin**	**Doxy-cycline**	**Cetirizine**	**Antiviral**	**Multi-vitamin**	**Other**
**Age (years)**										
18–20	1.000	1.000	1.000	1.000	1.000	1.000	1.000	1.000	1.000	1.000
21–25 and above	3.554^*^	1.331	2.766^*^	0.931	1.432	0.815	1.829^*^	2.918^*^	2.025^*^	1.172
**Gender**										
Female	1.136	1.220	0.723	1.078	1.289	0.321^*^	0.712	0.343^*^	2.573^*^	0.930
Male	1.000	1.000	1.000	1.000	1.000	1.000	1.000	1.000	1.000	1.000
**Student**										
Medical	1.001	1.022	0.965	1.078	1.048	1.159	0.636^*^	1.041	0.974	1.144
Pharmacy	1.000	1.000	1.000	1.000	1.000	1.000	1.000	1.000	1.000	1.000
**Medical year**										
1st year	1.000	1.000	1.000	1.000	1.000	1.000	1.000	1.000	1.000	1.000
2nd year	1.669	1.414	1.038	0.631	0.651	1.676	2.286^*^	1.105	0.909	1.155
3rd year	2.671^*^	2.063^*^	1.981^*^	0.757	2.475	3.543	2.056^*^	2.709	1.095	0.400
4th year	3.179^*^	2.210^*^	1.581	0.299	1.494	2.531	2.046	2.145	1.214	0.605
5th year	4.054^*^	1.255	3.219^*^	0.786	–	–	2.804^*^	1.551	1.366	0.284
**State**										
Sindh	1.000	1.000	1.000	1.000	1.000	1.000	1.000	1.000	1.000	1.000
Punjab	1.043	0.696	1.438	0.912	3.569^*^	1.770	0.617^*^	0.633	0.935	0.891
KPK/Balochistan	0.970	0.908	5.358^*^	1.564	2.812	2.962	1.468	1.570	1.953^*^	1.679
**Comorbidities**										
Present	0.760	0.913	0.850	0.761	2.235	1.188	0.972	1.315	0.776	7.627^*^
Absent	1.000	1.000	1.000	1.000	1.000	1.000	1.000	1.000	1.000	1.000
**Self-reported health**										
Excellent	1.000	1.000	1.000	1.000	1.000	1.000	1.000	1.000	1.000	1.000
Good	1.795^*^	1.022	1.365	0.826	2.989	1.294	1.299	0.294^*^	1.688^*^	2.143
Fair/Poor	1.559	0.947	1.447	1.055	3.784	3.041	1.611	0.512	1.523	3.524^*^
**COVID risk**										
No risk	1.000	1.000	1.000	1.000	1.000	1.000	1.000	1.000	1.000	1.000
Already infected	4.872^*^	0.935	5.795^*^	1.366	0.424	0.249	1.604	0.339	12.882^*^	0.888
Mild/Severe	2.446^*^	1.357	0.848	1.556	0.561	0.669	2.080^*^	1.150	1.758^*^	0.505^*^

*The dependent variable corresponds to the drug used while the reported p-values were obtained by logistic regression model, with the crude odds ratios reported*.

*P-values < 0.05 have a*

* *sign that indicates significant association with the dependent variable. Positive association implies to odds ratio >1.0 and negative association implies to odds ratio <1.0*.

*HCQ: hydroxychloroquine; KPK: Khyber Pakhtunkhwa*.

The following variables were significantly associated with self-medication practice in logistic regression (Supplementary Figure 1; Table 4): female sex (*p* < 0.001), third-year medical students (*p* = 0.022), self-reported health as good (*p* = 0.029), and those previously infected with COVID-19 (*p* = 0.050). In multivariate analysis, female gender (<0.001), those in third year of medical studies (*p* = 0.031) and those students who perceived their self-reported health as “good” were more likely to adopt self-medication practices (*p* = 0.032).

**Table 4:** Multivariate analysis of the factors associated with the self-medication practice during the COVID-19 lockdown among medical students (*n* = 489).

**Factors**	**OR**	**95% CI**	***p*-value**	**aOR**	**95% CI**	***p*-value**
**Age (years)**						
18–20	1.000	-	-	1.000	-	-
21–25 and above	1.091	0.674–1.767	0.722	1.106	0.656–1.865	0.706
**Gender**						
Female	2.547	1.531–4.239	<0.001^*^	2.810	1.630–4.843	<0.001^*^
Male	1.000	-	-	1.000	-	-
**Student**						
Medical	1.513	0.896–2.556	0.121	1.590	0.914–2.766	0.101
Pharmacy	1.000	-	-	1.000	-	-
**Medical year**						
1st year	1.000	-	-	1.000	-	-
2nd year	1.545	0.715–3.337	0.269	1.383	0.588–3.254	0.457
3rd year	3.003	1.175–7.677	0.022^*^	3.591	1.127–11.444	0.031^*^
4th year	1.655	0.669–4.093	0.276	1.517	0.435–5.289	0.513
5th year	1.094	0.453–2.644	0.842	1.034	0.278–3.849	0.960
**State**						
Sindh	1.000	-	-	1.000	-	-
Punjab	0.796	0.485–1.306	0.366	0.685	0.402–1.168	0.164
KPK and Balochistan	2.166	0.878–5.344	0.094	1.862	0.737–4.705	0.189
**Comorbidities**						
Present	0.526	0.200–1.388	0.195	0.567	0.202–1.590	0.281
Absent	1.000	-	-	1.000	-	-
**Self-reported health**						
Excellent	1.000	-	-	1.000	-	-
Good	1.826	1.065–3.132	0.029^*^	1.853	1.053–3.261	0.032^*^
Fair/Poor	1.576	0.756–3.286	0.225	1.530	0.710–3.299	0.278
**COVID risk**						
No risk	1.000	-	-	1.000	-	-
Already infected	2.230	0.999–4.980	0.050^*^	1.904	0.832–4.356	0.127
Mild/Severe	1.509	0.907–2.509	0.113	1.304	0.765–2.223	0.329

* *Indicates significant association with the dependent variable (P < 0.05)*.

*KPK, Khyber Pakhtunkhwa; COVID, coronavirus disease*.

## Discussion

Self-medication is often considered as an ambiguous phenomenon and the practice is commonplace in developed and developing countries alike. However, it has not been extensively qualitatively or quantitatively studied, especially in Pakistan (20). Self-medication with antibiotics rates were generally low in middle income countries, according to a 2020 review, however, among affected countries, Pakistan had one of the highest prevalence of 81.23%, with only 7.3% in Indonesia and 26.9% in Bangladesh (21). A study conducted by the Aga Khan University, Karachi in 2008 revealed 76% self-medication rates among the medical and non-medical university students surveyed (22), while another survey from pre-COVID times, consisting of a small segment of medical students in Pakistan found 99% of them to be engaged in practicing self-medication (23). To the best of our knowledge, our study is the first of its kind within the current pool of literature available, that has evaluated self-medication among medical students in the context of COVID-19, encompassing most provinces of Pakistan.

The incidence of self-medication revealed in our results is around 83%, a finding that is comparable to self-medication occurrence rates during COVID-19 reported in studies conducted abroad. Among the nursing undergraduates in Saudi Arabia, self-medication practice with antibiotics and analgesics was found to be 87% (5) while In Kenya, health care self-medication prevalence was 60.4% among healthcare workers, indicating an increase from pre pandemic results (24). Another study assessing self-medication prevalence in the Nigerian population during the pandemic reported a finding of 41% (25). In Togo, 34.1 % of the participants that belonged to healthcare, air transport, police, road transport and informal sectors reported to self-medicate and healthcare division had the highest of 51.9% against all sectors (26). Conversely, in Peru, the number of responders not engaging in self-medication was higher than those that did, for all drugs including Acetaminophen, Ibuprofen, Azithromycin, Hydroxychloroquine, Penicillin, Antiretrovirals, which were evaluated (16). Multivariate logistic regression model showed that being female gender, medical school year 3rd, having been already infected with COVID-19 or having already infected with COVID-19 were factors that were associated respondents expected to self-medicate the most.

Paracetamol was the highest used drug in our study, respondents 21–25 years and above, 5th year medical students, those already infected with COVID-19, and stating symptoms of cold/flu, fever and fatigue showed significant use. High use of Paracetamol aligns with results from the nursing undergraduates in Saudi Arabia (17) and from the Peru adult population (16) where it was also the most consumed drug. As a widely used first line pharmacotherapy analgesic for tackling pain disorders and different pyrexia, Paracetamol use is widely reported in studies comprising of medical students. Health science major students in Nigeria commonly used it as a pain reliever (25), while medical students in Iran were also frequent users of this drug (27). An Indian study found medical students to be more likely to self-medicate than paramedical students and for this purpose, Paracetamol was consumed mostly (28). Paracetamol has been known to have negligible anti-inflammatory action (28) and guidelines issued during the initial months of the pandemic state that it can be used for treating mild COVID-19 symptoms when managing patients at home (29) or for self-medication, especially for fever or headache (30). This is consistent with our results corresponding to Paracetamol use as it was greatly used to treat “fever” in our study population, and its use also showed improvement in all or most of the symptoms experienced by the responders who employed Paracetamol. However, some instances report the drug to be exacerbating COVID-19 symptoms, likely due to activation of prothrombotic mechanisms which is known as one of the leading pathogenic causes of COVID-19 (29). Single or repeated high Paracetamol dose, or chronic ingestion above therapeutic doses can result in hepatotoxicity (17). Hepatotoxicity from intentional or non-intentional Paracetamol overdose is the most common cause of drug-induced liver injury and is a global issue (17). The medicine's toxic dosage effects must be known to students utilizing it.

Multivitamins were the second highest consumed drug in our study, which have also been the drug of choice as divulged in a population-based survey during COVID-19 in Nigeria with 51.8% reporting to use this group of medicine (25). In Togo, 27.6% used Vitamin C when self-medicating (26), while in Egypt, 27% used vitamin C and 17.7% used vitamin D (31). A study from 2015 involving healthcare and non-healthcare university students from South India found multivitamins to be among the most commonly self-medicated drugs (32). Multivitamins include multiple vitamins and minerals, trace elements all of which possess antioxidant properties and have a crucial role in regulating immune function and can reduce risk of respiratory infection (33). A large observational study conducted in US, UK, and Sweden during first waves of COVID-19 demonstrated a modest protective effect among those taking multivitamin supplements with a reduced risk for testing positive for SARS-CoV-2 (33). Hence, these might explain the significant use of multivitamins by females and participants 21–25 years and above. Additionally, individuals with COVID-19 can experience weight loss; having adequate amounts of vitamins and minerals can prevent this unintended reduction in weight (34). Moreover, a deficiency in certain micronutrients such as Vitamin A, Vitamin D, Zinc can be deleterious during viral infections (33). These factors might elucidate why those infected with COVID-19 in our research resorted to a significantly high rate of multivitamin use.

Ibuprofen was the third highest self-medicated drug in our population. Previously, it has been reported to be the most preferred NSAID for analgesic purposes among nursing undergraduates in Saudi Arabia (20%) (17). As a non-steroidal anti-inflammatory drug (NSAID), it is well tolerated and effective during pain and inflammation and has minor side effects (17). Alongside Paracetamol, it is one of the most extensively used antipyretic (35). Since the start of the pandemic, Ibuprofen use has been subject to debate (36). Initially discouraged for use as it was suspected to aggravate infections (37), such claims were later rejected. A study analyzing data from ISARIC Clinical Characterization Protocol UK cohort found NSAIDS use not disposing to any poor COVID-19 outcome (38). A 2021 systematic review and meta-analysis reiterated similar findings and revealed no negative association between use of NSAIDS, including Ibuprofen, with SARS-CoV-2 infection or its outcomes (39). Ibuprofen has been known to have a better analgesic response in men while higher threshold and greater tolerance for electrically induced pain, responded significantly to ibuprofen in contrast to women (40). Nevertheless, the high use of this drug during COVID-19 can be linked to its efficacy in relieving fever, infection (35). However, users must be wary that disproportionate use of the drug can cause adverse drug reactions (ADRs) (17).

Hydroxychloroquine and ivermectin, the sales of which escalated globally in wake of COVID-19 were found to be consumed in low amounts in our study (41). Other frequently used drugs were Cetirizine and Azithromycin. Azithromycin has been popular amongst antibiotics that have been used for self-medication in Pakistan (21) and has also been an alternative for self-medication in other countries such as Saudi Arabia (17) and Peru (16). Using antibiotics during self-medication is the leading cause of antimicrobial resistance (42) thus its intake must be regulated, its appropriate use and disposal ensured especially by medical students.

The most common reason for use of the respective drug was “cold/flu”, consistent with results from studies of including medical students from Iran (29, 43) and Brazil (44). “Fever”, one of the most common COVID-19 symptom (19), followed by “Muscle Pain” were the highest symptomologies reported for medicine use. Analgesic use for muscular pain at any site was also highly common among undergraduate medical and paramedical students in India (28). Studies evaluating medical students' attitudes toward self-medication delineate sufficient knowledge, familiarity with disease, and time constraints which can also be cited as motives for self-medication among our participants in this pandemic scenario (17). In the early days of SARS-CoV-2 outbreak, when the disease was a source of fear and stigma, knowledge and access to prescription-only medicines while trying to keep one's health status, especially if infected with COVID-19, could be accounted for self-medication rates among the medical students' community (26). Furthermore, practicing self-medication allowed the students to exercise freedom in managing their health and wellbeing instilling a sense of independence (24). The overall high self-medication rate throughout all year groups can be ascribed to the extensive educational load upon these students and stressful circumstances which results in distress and compels them to seek OTC drugs (45). Another study conducted in Poland highlighted such behaviors increased during lockdown (46). According to a study from Jordan, factors such as female gender, working in the medical field, and history of COVID-19 infection were reportedly associated with self-medication similar to our findings (47).

While the high self-medication percentage suggests medical students' confidence while self-prescribing, the possibility that students' knowledge about drug pharmacology, cultural prevalence, and limited experience at the current student level can make them susceptible to unfavorable outcomes, must not be undermined (28). Drug addiction and drug dependence (29) can also be induced by repeated self-medication, which students must be aware of. Lockdown and isolation guidelines have prevented the population from visiting hospitals and clinics and have altered health-seeking behavior. As different SARS-CoV-2 variants keep emerging, people are likely to self-medicate (48). Medical students are also probable to keep self-medicating, which could be helpful in obtaining benefits of self-care, and alleviating burden on the healthcare system of the country by reducing number of physician visits (49). By practicing self-medication wisely themselves, staying mindful of the pharmacological and taxological risks of improper drug use (25), they could facilitate the lay population to treat themselves for minor ailments which could further relieve pressure on health care setups.

The study is important as questions specific to the drug trends in the local population were incorporated, and responses from all parts of the country were received. However, as online platforms were used for disseminating the questionnaire, students in remote areas with no access to the internet could not be a part of the survey. Other limitation included non-random sampling method and generalizability of study results. Besides, due to recall bias, some responders might have answered incorrectly.

## Conclusion

Our study revealed common self-medication practices among medical and pharmacy students. It is a significant health issue especially during the pandemic times, with high consumption reported as a prevention or treating symptoms of COVID-19. Further measures are needed to improve healthcare policies regarding awareness and sensitization about the risks of self-medication. Future studies should also assess students' attitude toward self-medication, their knowledge regarding drug dosage and potential side effects, and the role of medical colleges to better ascertain their approach toward self-medication.

## Data Availability Statement

The raw data supporting the conclusions of this article will be made available by the authors, without undue reservation.

## Ethics Statement

The studies involving human participants were reviewed and approved by Ethics Approval was taken in this study from Institutional Review Board of Dow University Ojha Hospital (Approval No. IRB (DUH)-2020/174/021), and content to participate was obtained from all the individuals through online platform. The patients/participants provided their written informed consent to participate in this study.

## Author Contributions

FY: conceptualization. UN, HNaj, HNau, and AK: data curation. MSA: formal analysis. FY and MNA: funding acquisition. FY, UN, and HNaj: investigation. MSA and AK: methodology. FY, UN, and AK: project administration. UN, HNaj, and HNau: resources. MSA and HNau: software. MSA, HNau, and AK: validation. HNaj and HNau: visualization. UN and HNau: writing—original draft. FY, MSA, MNA, and AK: writing—review and editing. All authors approved the final version of the manuscript.

## Conflict of Interest

The authors declare that the research was conducted in the absence of any commercial or financial relationships that could be construed as a potential conflict of interest.

## Publisher's Note

All claims expressed in this article are solely those of the authors and do not necessarily represent those of their affiliated organizations, or those of the publisher, the editors and the reviewers. Any product that may be evaluated in this article, or claim that may be made by its manufacturer, is not guaranteed or endorsed by the publisher.

## Supplementary Material

Supplementary Figure 1 Crude and Adjusted level analysis of factors associated with self-medication among medical students.
