# Supplementary figures and images for: Self-Medication Practices in Medical Students During the COVID-19 Pandemic: A Cross-Sectional Analysis
Source: Front Public Health. 2022 Mar 9;10:803937. doi: 10.3389/fpubh.2022.803937 (PMC8959567; doi:10.3389/fpubh.2022.803937)

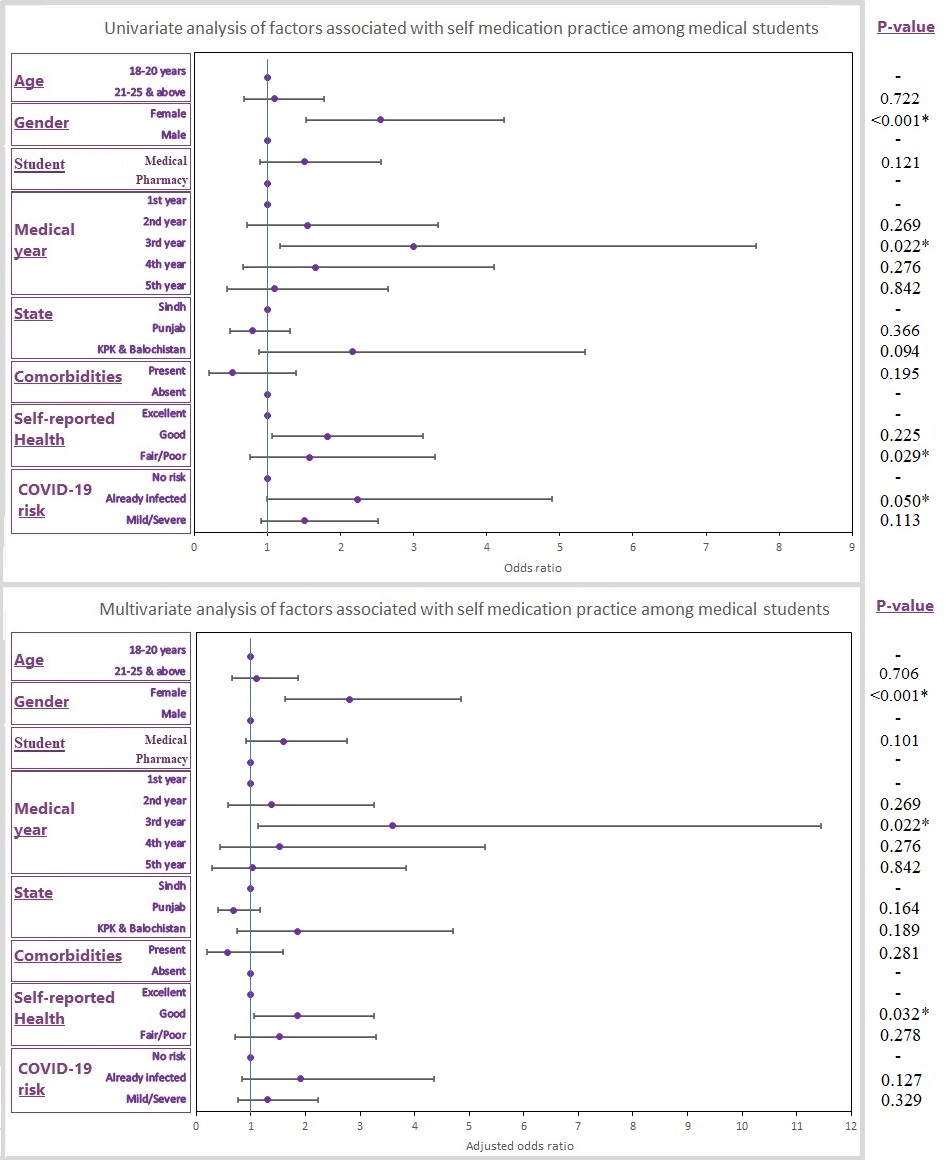

Supplement: Supplementary Figure 1 — Crude and Adjusted level analysis of factors associated with self-medication among medical students. [file Image_1.TIFF]
